# Trends and outcomes of blunt renal trauma management: a nationwide cohort study in Japan

**DOI:** 10.1186/s13017-020-00329-w

**Published:** 2020-08-26

**Authors:** Shunichiro Nakao, Yusuke Katayama, Atsushi Hirayama, Tomoya Hirose, Kenichiro Ishida, Yutaka Umemura, Jotaro Tachino, Takeyuki Kiguchi, Tasuku Matsuyama, Kosuke Kiyohara, Tetsuhisa Kitamura, Yuko Nakagawa, Takeshi Shimazu

**Affiliations:** 1 Department of Traumatology and Acute Critical Medicine, Osaka University Graduate School of Medicine, 2-15 Yamadaoka, Suita, Suita, Osaka 565-0871 Japan; 2 Public Health, Department of Social Medicine, Graduate School of Medicine, Osaka University, Suita, Japan; 3 Department of Acute Medicine and Critical Care Medical Center, Osaka National Hospital, National Hospital Organization, Osaka, Japan; 4 Department of Emergency and Critical Care, Osaka General Medical Center, Osaka, Japan; 5 Kyoto University Health Service, Kyoto, Japan; 6 Department of Emergency Medicine, Kyoto Prefectural University of Medicine, Kyoto, Japan; 7 Department of Food Science, Faculty of Home Economics, Otsuma Women’s University, Tokyo, Japan; 8 Division of Environmental Medicine and Population Sciences, Department of Social and Environmental Medicine, Graduate School of Medicine, Osaka University, Suita, Japan

**Keywords:** Renal trauma, Blunt injury, Nephrectomy, Japan Trauma Data Bank

## Abstract

**Background:**

There is a paucity of information for predicting patient outcomes other than the American Association for the Surgery of Trauma (AAST) renal injury scale. The aim of this study was to evaluate the association between the patient characteristics and outcomes of patients with blunt renal trauma using a nationwide database in Japan.

**Methods:**

We performed a retrospective analysis of the Japan Trauma Data Bank (JTDB) from 2004 to 2018. We identified patients with blunt renal trauma by AIS codes converted to AAST grades. We evaluated trends in patient characteristics and management and assessed factors associated with mortality and nephrectomy using a multivariable logistic regression analysis.

**Results:**

We identified 3550 patients with blunt renal trauma. Their median age was 43 years and 74.2% were male. Nephrectomy was performed in 3.8%, and the overall mortality rate was 9.5%. We found increasing trends in age and emergency abdominal angiography and decreasing trends in nephrectomy and mortality over the 15-year period. The following factors were associated with mortality: age ≥ 65 years (adjusted OR 3.36); pedestrian accident (adjusted OR 1.94); fall from height (adjusted OR 1.91); shock on arrival (adjusted OR 4.02); concomitant injuries to the head/neck (adjusted OR 3.14), pelvis/lower-extremity (adjusted OR 1.59), liver (adjusted OR 1.68), spleen (adjusted OR 1.45), and gastrointestinal tract (adjusted OR 1.90); AAST grades III–V (adjusted ORs 1.42, 2.16, and 5.55); and emergency abdominal angiography (adjusted OR 0.70). The following factors were associated with nephrectomy: shock on arrival (adjusted OR 1.98), concomitant injuries to the thorax (adjusted OR 0.46) and spleen (adjusted OR 2.07), AAST grades III, IV, and V (adjusted ORs 18.40, 113.89, and 468.17), and emergency abdominal angiography (adjusted OR 0.28).

**Conclusions:**

We demonstrated that the AAST grade and emergency angiography were associated with mortality and nephrectomy in blunt renal trauma in the Japanese population.

## Background

Renal trauma, which accounts for 1–5% of all trauma and up to 10% of abdominal trauma, is predominantly caused by blunt mechanisms of injury [1, 2]. A previous systematic review in 2005 noted that non-operative management of renal trauma was not yet universally accepted, despite the fact that many studies supported non-operative management [3]. However, more recent systematic reviews have reported favorable outcomes of non-operative management, even in high-grade renal trauma [4, 5]. In recent years, less invasive interventions, such as endovascular procedures, have increasingly been used for blunt renal trauma [6, 7]. Nevertheless, nephrectomy is still required for unstable patients those in whom non-operative management fails [8–10]. According to a report by van der Wilden et al., among patients injured by road traffic accidents, non-operative management failed in 27.3% of the patients who were > 55 years of age [11]. It is important to identify patients who require nephrectomy after renal trauma.

While multiple studies have shown the efficacy and safety of non-operative management, there is still a paucity of information on the characteristics of blunt renal trauma and current managements and their trends. Furthermore, there is little evidence on predictors of the need for nephrectomy other than the American Association for the Surgery of Trauma (AAST) renal injury scale [12]. The aim of this study was to evaluate the association between patient characteristics and outcomes such as mortality and the need for nephrectomy in patients with blunt renal trauma using a nationwide database in Japan, in considering trends in management.

## Methods

### Study design and setting

We performed a retrospective analysis of the Japan Trauma Data Bank (JTDB). The institutional ethics committee of Osaka University Graduate School of Medicine approved this study and waived the requirement for informed consent because all of the analyses used anonymous data (approval no. 16260).

### Japan Trauma Data Bank

The JTDB is a nationwide voluntary hospital-based trauma registry that was established in 2003 by the Japanese Association for the Surgery and Trauma (Trauma Surgery Committee) and the Japanese Association for Acute Medicine (Committee for Clinical Care Evaluation) [13]. In 2018, 280 major emergency medical institutions across Japan participated in the JTDB registry [14]. The ability of these hospitals is equivalent to that of level I trauma centers in the USA. Data were collected from participating institutions via the internet. In most cases, physicians and medical assistants who completed the Abbreviated Injury Scale (AIS) coding course registered the patients’ data.

The JTDB captures the following data in trauma cases: age, sex, mechanism of injury, AIS code (version 1998), Injury Severity Score (ISS), vital signs on hospital arrival, date and time series from hospital arrival to discharge, medical managements (e.g., interventional radiology), surgical operations and computed tomography scanning, complications, and mortality at discharge. The ISS was calculated from the top three scores of the AIS in the nine anatomical regions classified by the AIS code.

### Participants

The cases of patients who were admitted in the years 2004 to 2018 and whose information was registered in the JTDB were analyzed. We included blunt trauma patients with traumatic renal injuries, which were identified by AIS codes using the method described by Kuan et al. [12] AIS codes were converted to AAST renal injury grades, excluding codes that did not match [15–17]. We excluded patients who were in cardiac arrest on hospital arrival, and those whose records were missing information on age, sex, vital signs on arrival, ISS, or mortality. We defined cardiac arrest on hospital arrival as a systolic blood pressure of 0 mmHg or a heart rate of 0 bpm on hospital arrival.

### Variables

We extracted the following patient data from the JTDB database: age, sex, mechanism of injury, AIS code, ISS, vital signs on hospital arrival, interventions (e.g., emergency abdominal angiography or nephrectomy), and mortality at discharge. To evaluate temporal trends, we divided the 15-year study period into five periods: 2004–2006, 2007–2009, 2010–2012, 2013–2015, and 2016–2018. We categorized age into three groups: < 20 years, 20–64 years, and ≥ 65 years. We defined shock as a systolic blood pressure of < 80 mmHg on hospital arrival [18]. To assess concomitant injuries, we mapped AIS-coded injuries to the following categories: head/neck, thorax, pelvis/extremities, and intra-abdominal organs (including the liver, spleen, pancreas, and gastrointestinal tract).

### Statistical analyses

Continuous variables are presented as the median and interquartile range (IQR), and categorical variables are presented as the number and percentage. The Jonckheere-Terpstra test was used to analyze trends in continuous variables, and the Cochrane-Armitage test was used to analyze trends in categorical variables.

Factors associated with mortality were assessed by a multivariable logistic regression analysis, and adjusted odds ratios (ORs) and 95% confidence intervals (CIs) were calculated. A multivariable logistic regression analysis was performed with a forced entry procedure. The independent parameters included age group (< 20 years, 20–64 years, ≥ 65 years), sex, mechanism of injury, shock on arrival, each concomitant injury, AAST renal injury grade, and interventions (e.g., emergency abdominal angiography or nephrectomy), and the 3-year time period. We also assessed factors associated with nephrectomy using a multivariable logistic regression analysis. The fit of the models was evaluated with the Hosmer-Lemeshow goodness-of-fit test.

As a further analysis, we divided patients into those with isolated renal trauma and those with multiple trauma to evaluate the difference in patient demographics. The patient characteristics were compared between the groups using the Mann-Whitney *U* test for continuous variables and the chi-squared test for categorical variables.

All tests were two-tailed, and *P* values of < 0.05 were considered to indicate statistical significance. All statistical analyses were performed using R Statistical Software (version 3.6.2; R Foundation for Statistical Computing, Vienna, Austria).

## Results

**Fig. 1:**
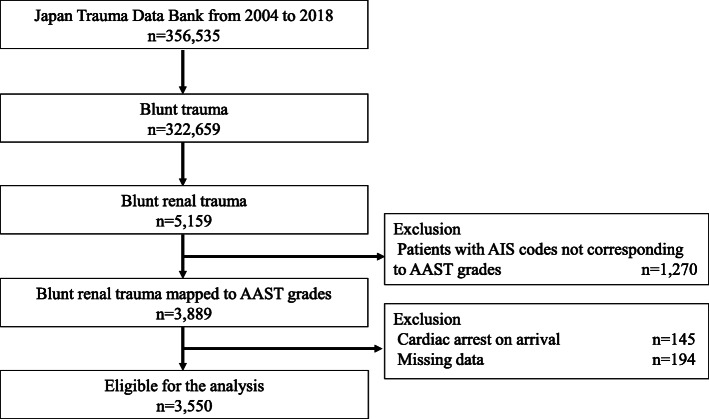
Patient flow

Figure 1 shows the patient flow of the study. During the study period, 356,535 patients were recorded in the JTDB database and 322,659 patients had blunt injuries. There were 5159 patients with blunt renal trauma. Among them, 3550 (1.0%) patients with renal trauma that could be converted to an AAST grade from AIS codes were eligible for inclusion in the analysis. The patient characteristics and their temporal trends are summarized in Table 1. The median age of the overall patient population was 43 years (IQR, 23–65 years), 17.1% were younger than 20 years of age, 26.1% were 65 years of age or older, and 74.2% of the patients were male. The most frequent mechanism of injury was motorcycle accident (22.5%), followed by fall from height (17.4%) and car accident (15.7%). The median ISS was 22 (IQR, 14–34), and 11.4% were in shock on arrival at the hospital. Emergency abdominal angiography was performed in 33.5% of the patients, while nephrectomy was performed in 3.8%. The overall mortality rate in the cohort was 9.5%.

**Table 1:** Patient characteristics of patients with blunt renal trauma and temporal trends from 2004 to 2018

Characteristics	Total		2004–2006		2007–2009		2010–2012		2013–2015		2016–2018		*P* for trend
	*n* = 3550		*n* = 189		*n* = 451		*n* = 819		*n* = 1111		*n* = 980		
Age, median, Q1–Q3	43	23–65	29	20–52	37	21–60	42	23–64	44	24–66	48	25–69	0.002
Age group, *n* (%)													
< 20 years	607	(17.1)	36	(19.0)	89	(19.7)	140	(17.1)	186	(16.7)	156	(15.9)	0.076
20–64 years	2017	(56.8)	128	(67.7)	271	(60.1)	479	(58.5)	631	(56.8)	508	(51.8)	< 0.001
≥ 65 years	926	(26.1)	25	(13.2)	91	(20.2)	200	(24.4)	294	(26.5)	316	(32.2)	< 0.001
Male sex, *n* (%)	2635	(74.2)	138	(73.0)	324	(71.8)	582	(71.1)	843	(75.9)	748	(76.3)	0.014
Mechanism, *n* (%)													
Car accident	556	(15.7)	37	(19.6)	62	(13.7)	136	(16.6)	174	(15.7)	147	(15.0)	0.448
Motorcycle accident	797	(22.5)	46	(24.3)	129	(28.6)	188	(23.0)	244	(22.0)	190	(19.4)	< 0.001
Bicycle accident	271	(7.6)	9	(4.8)	38	(8.4)	65	(7.9)	78	(7.0)	81	(8.3)	0.486
Pedestrian accident	353	(9.9)	22	(11.6)	42	(9.3)	82	(10.0)	125	(11.3)	82	(8.4)	0.337
Fall from height	616	(17.4)	38	(20.1)	71	(15.7)	147	(17.9)	195	(17.6)	165	(16.8)	0.675
Fall down stairs	323	(9.1)	8	(4.2)	25	(5.5)	74	(9.0)	102	(9.2)	114	(11.6)	< 0.001
Fall on the ground	265	(7.5)	6	(3.2)	30	(6.7)	54	(6.6)	92	(8.3)	83	(8.5)	0.008
Sports-related injury	145	(4.1)	10	(5.3)	12	(2.7)	26	(3.2)	38	(3.4)	59	(6.0)	0.018
Others	224	(6.3)	13	(6.9)	42	(9.3)	47	(5.7)	63	(5.7)	59	(6.0)	0.092
ISS, median, Q1–Q3	22	14-34	22	16-34	22	16-34	22	15-34	22	14-34	22	14-34	0.072
Shock on arrival, *n* (%)	405	(11.4)	27	(14.3)	64	(14.2)	102	(12.5)	123	(11.1)	89	(9.1)	< 0.001
AAST grade													
I	1408	(39.7)	88	(46.6)	172	(38.1)	326	(39.8)	457	(41.1)	365	(37.2)	0.139
II	358	(10.1)	21	(11.1)	47	(10.4)	76	(9.3)	99	(8.9)	115	(11.7)	0.506
III	1039	(29.3)	39	(20.6)	122	(27.1)	229	(28.0)	336	(30.2)	313	(31.9)	< 0.001
IV	596	(16.8)	32	(16.9)	83	(18.4)	147	(17.9)	182	(16.4)	152	(15.5)	0.150
V	149	(4.2)	9	(4.8)	27	(6.0)	41	(5.0)	37	(3.3)	35	(3.6)	0.021
Concomitant injury, *n* (%)													
Head/neck	1341	(37.8)	88	(46.6)	182	(40.4)	313	(38.2)	419	(37.7)	339	(34.6)	0.001
Thorax	2298	(64.7)	109	(57.7)	280	(62.1)	530	(64.7)	726	(65.3)	653	(66.6)	0.013
Pelvis/lower-extremity	1399	(39.4)	93	(49.2)	179	(39.7)	329	(40.2)	438	(39.4)	360	(36.7)	0.008
Concomitant intra-abdominal organ injury, *n* (%)													
Liver	972	(27.3)	57	(30.2)	130	(28.8)	225	(27.5)	314	(28.3)	246	(25.1)	0.094
Spleen	655	(18.5)	41	(21.7)	88	(19.5)	145	(17.7)	207	(18.6)	174	(17.8)	0.287
Pancreas	78	(2.2)	4	(2.1)	9	(2.0)	19	(2.3)	20	(1.8)	26	(2.7)	0.573
Gastrointestinal tract	98	(2.8)	5	(2.6)	10	(2.2)	25	(3.1)	27	(2.4)	31	(3.2)	0.532
Isolated traumatic renal injury, *n* (%)	509	(14.3)	20	(10.6)	62	(13.7)	119	(14.5)	151	(13.6)	157	(16.0)	0.088
Management, *n* (%)													
Emergency abdominal angiography	1,189	(33.5)	49	(25.9)	127	(28.2)	275	(33.6)	390	(35.1)	348	(35.5)	< 0.001
Nephrectomy	136	(3.8)	10	(5.3)	39	(8.6)	36	(4.4)	27	(2.4)	24	(2.4)	< 0.001
Mortality, *n* (%)	337	(9.5)	29	(15.3)	56	(12.4)	90	(11.0)	90	(8.1)	72	(7.3)	< 0.001

*P* values for trend were calculated using the Jonckheere-Terpstra test and Cochrane-Armitage test

*ISS* Injury Severity Score, *AAST* American Association for the Surgery of Trauma

Over 15 years, the median age increased from 29 years to 48 years old (*P* for trend = 0.002). The proportion of patients of 20 to 64 years of age was decreased (*P* for trend < 0.001), while that of patients of ≥ 65 years of age was significantly increased (*P* for trend < 0.001). The percentage of male patients increased from 73.0 to 76.3% (*P* for trend = 0.014). The percentage of victims of motorcycle accidents decreased (*P* for trend < 0.001), while the percentages of patients with falls down stairs, falls on the ground, and sports-related injury increased (*P* for trend < 0.001, = 0.008, and = 0.018, respectively). The percentage of patients who were in shock on hospital arrival was decreased (*P* for trend < 0.001). Regarding the distribution of the AAST renal injury grades, there was a significant increase in patients with AAST grade III (*P* for trend < 0.001) and a significant decrease in patients with AAST grade V (*P* for trend = 0.008). There was a decrease in concomitant head/neck injury (*P* for trend = 0.001) and concomitant pelvis/lower-extremity injury (*P* for trend = 0.008), and there was an increase in concomitant thoracic injury (*P* for trend = 0.013). There was a significant increase in emergency abdominal angiography from 25.9% in 2004–2006 to 35.5% in 2016–2018 (*P* for trend < 0.001). Meanwhile, there was a corresponding decrease in nephrectomy from 5.3 to 2.4% (*P* for trend < 0.001). Mortality declined significantly from 15.3% in 2004–2006 to 7.3% in 2016–2018 (*P* for trend < 0.001).

Table 2 summarizes the associations between mortality and various factors. Age ≥ 65 years (adjusted OR 3.36 [95% CI 2.16 to 5.34]), pedestrian accident (adjusted OR 1.94 [95% CI 1.26 to 3.07]), fall from height (adjusted OR 1.91 [95% CI 1.25 to 2.96]), shock on hospital arrival (adjusted OR 4.02 [95% CI 3.01 to 5.34]), concomitant head/neck injury (adjusted OR 3.14 [95% CI 2.39 to 4.14]), concomitant pelvis/lower-extremity injury (adjusted OR 1.59 [95% CI 1.21 to 2.08]), concomitant liver injury (adjusted OR 1.68 [95% CI 1.27 to 2.21]), concomitant splenic injury (adjusted OR 1.45 [95% CI 1.06 to 1.97]), concomitant gastrointestinal tract injury (adjusted OR 1.90 [95% CI 1.01 to 3.55]), and AAST grade III, IV, and V (adjusted ORs 1.42 [95% CI 1.02 to 1.96], 2.16 [95% CI 1.48 to 3.13], and 5.55 [95% CI 3.22 to 9.49], respectively) were associated with higher mortality. Emergency abdominal angiography was associated with lower mortality (adjusted OR 0.70 [95% CI 0.53 to 0.93]), but nephrectomy was not. This model had a non-significant Hosmer-Lemeshow goodness-of-fit statistic (*P* = 0.390).

**Table 2:** Odds ratios of each variable for mortality among patients with blunt renal trauma

	Mortality			
	%	*n*/*N*	Adjusted OR (95% CI)	*P* value
Age group				
< 20 years	5.3	(32/607)	Reference	–
20–64 years	8.5	(171/2017)	1.45 (0.96 to 2.23)	0.084
≥ 65 years	14.5	(134/926)	3.36 (2.16 to 5.34)	< 0.001
Sex				
Male	8.8	(233/2635)	0.96 (0.72 to 1.28)	0.759
Female	11.4	(104/915)	Reference	–
Mechanism				
Car accident	8.1	(45/556)	Reference	–
Motorcycle accident	9.3	(74/797)	1.42 (0.92 to 2.21)	0.120
Bicycle accident	9.6	(26/271)	1.51 (0.86 to 2.62)	0.147
Pedestrian accident	19.8	(70/353)	1.94 (1.26 to 3.07)	0.004
Fall from height	14.1	(87/616)	1.91 (1.25 to 2.96)	0.003
Fall down stairs	3.1	(10/323)	0.55 (0.25 to 1.13)	0.122
Fall on the ground	2.3	(6/265)	0.66 (0.24 to 1.58)	0.384
Sports-related injury	0.7	(1/145)	0.47 (0.03 to 2.32)	0.463
Others	8.0	(18/224)	1.31 (0.69 to 2.41)	0.389
Shock on arrival				
(+)	31.4	(127/405)	4.02 (3.01 to 5.34)	< 0.001
(−)	6.7	(210/3145)	Reference	–
Concomitant injury				
Head/neck				
(+)	16.9	(226/1341)	3.14 (2.39 to 4.14)	< 0.001
(−)	5.0	(111/2209)	Reference	–
Thorax				
(+)	11.8	(271/2298)	1.33 (0.97 to 1.86)	0.084
(−)	5.3	(66/1252)	Reference	–
Pelvis/lower-extremity				
(+)	15.1	(211/1399)	1.59 (1.21 to 2.08)	0.001
(−)	5.9	(126/2151)	Reference	–
Concomitant intra-abdominal organ injury				
Liver				
(+)	12.9	(125/972)	1.68 (1.27 to 2.21)	< 0.001
(−)	8.2	(212/2578)	Reference	–
Spleen				
(+)	12.5	(82/655)	1.45 (1.06 to 1.97)	0.018
(−)	8.8	(255/2,895)	Reference	–
Pancreas				
(+)	11.5	(9/78)	0.66 (0.28 to 1.41)	0.307
(−)	9.4	(328/3472)	Reference	–
Gastrointestinal tract				
(+)	18.4	(18/98)	1.90 (1.01 to 3.55)	0.046
(−)	9.2	(319/3452)	Reference	–
Isolated renal trauma				
(+)	1.4	(7/509)	0.66 (0.25 to 1.50)	0.348
(−)	10.9	(330/3041)	Reference	–
AAST grade				
I	8.5	(119/1408)	Reference	–
II	7.8	(28/358)	0.89 (0.55 to 1.40)	0.633
III	7.8	(81/1039)	1.42 (1.02 to 1.96)	0.036
IV	11.1	(66/596)	2.16 (1.48 to 3.13)	< 0.001
V	28.9	(43/149)	5.55 (3.22 to 9.49)	< 0.001
Management				
Emergency abdominal angiography	8.7	(103/1189)	0.70 (0.53 to 0.93)	0.015
(+)	9.9	(234/2361)	Reference	–
(−)				
Nephrectomy				
(+)	23.5	(32/136)	0.97 (0.54 to 1.68)	0.905
(−)	8.9	(305/3414)	Reference	–
3-year increase in time period	–	–	0.83 (0.75 to 0.93)	0.001

*OR* odds ratio, *CI* confidence interval, *ISS* Injury Severity Score, *AAST* American Association for the Surgery of Trauma

Table 3 summarizes the associations between nephrectomy and various factors. Nephrectomy was significantly associated with shock on hospital arrival (adjusted OR 1.98 [95% CI 1.21 to 3.20]) and concomitant splenic injury (adjusted OR 2.07 [95% CI 1.27 to 3.33]). AAST grades III, IV, and V (adjusted ORs 18.40 [95% CI 5.31 to 115.88], 113.89 [95% CI 34.83 to 701.57], and 468.17 [95% CI 137.15 to 2941.20], respectively) were positively associated with nephrectomy, while concomitant thoracic injury (adjusted OR 0.46 [95% CI 0.29 to 0.75]) and emergency abdominal angiography (adjusted OR 0.28 [95% CI 0.18 to 0.44]) were negatively associated with nephrectomy. This model demonstrated good fit in a Hosmer-Lemeshow goodness-of-fit test (*P* = 0.863).

**Table 3:** Odds ratios of each variable for nephrectomy among patients with blunt renal trauma

	Nephrectomy			
	%	*n*/*N*	Adjusted OR (95% CI)	*P* value
Age group				
< 20 years	3.5	(21/607)	Reference	–
20–64 years	4.3	(87/2017)	1.33 (0.77 to 2.38)	0.327
≥ 65 years	3.0	(28/926)	1.37 (0.68 to 2.78)	0.376
Sex				
Male	3.6	(94/2635)	0.81 (0.51 to 1.28)	0.354
Female	4.6	(42/915)	Reference	–
Mechanism				
Car accident	3.2	(18/556)	Reference	–
Motorcycle accident	4.9	(39/797)	1.10 (0.56 to 2.25)	0.782
Bicycle accident	2.2	(6/271)	0.89 (0.30 to 2.39)	0.832
Pedestrian accident	4.8	(17/353)	1.25 (0.54 to 2.89)	0.599
Fall from height	4.7	(29/616)	1.40 (0.69 to 2.93)	0.359
Fall down stairs	2.2	(7/323)	0.43 (0.14 to 1.14)	0.101
Fall on the ground	2.3	(6/265)	0.49 (0.16 to 1.37)	0.189
Sports-related injury	1.4	(2/145)	0.32 (0.05 to 1.30)	0.156
Others	5.4	(12/224)	0.99 (0.40 to 2.40)	0.981
Shock on arrival				
(+)	11.1	(45/405)	1.98 (1.21 to 3.20)	0.006
(−)	2.9	(91/3145)	Reference	–
Concomitant injury				
Head/neck				
(+)	3.3	(44/1341)	0.87 (0.54 to 1.39)	0.556
(−)	4.2	(92/2209)	Reference	–
Thorax				
(+)	3.2	(74/2298)	0.46 (0.29 to 0.75)	0.002
(−)	5.0	(62/1252)	Reference	–
Pelvis/lower-extremity				
(+)	3.8	(53/1399)	1.00 (0.63 to 1.59)	0.998
(−)	3.9	(83/2151)	Reference	–
Concomitant intra-abdominal organ injury				
Liver				
(+)	4.0	(39/972)	1.37 (0.84 to 2.21)	0.197
(−)	3.8	(97/2578)	Reference	–
Spleen				
(+)	6.7	(44/655)	2.07 (1.27 to 3.33)	0.003
(−)	3.2	(92/2895)	Reference	–
Pancreas				
(+)	9.0	(7/78)	0.74 (0.25 to 1.90)	0.545
(−)	3.7	(129/3472)	Reference	–
Gastrointestinal tract				
(+)	10.2	(10/98)	1.14 (0.45 to 2.70)	0.771
(−)	3.7	(126/3452)	Reference	–
Isolated renal trauma				
(+)	3.9	(20/509)	0.84 (0.41 to 1.73)	0.642
(−)	4.1	(126/3041)	Reference	–
AAST grade				
I	0.1	(2/1408)	Reference	–
II	0	(0/358)	0.00 (0.00 to 0.00)	0.981
III	1.9	(20/1039)	18.40 (5.31 to 115.88)	< 0.001
IV	10.2	(61/596)	113.89 (34.83 to 701.57)	< 0.001
V	35.6	(53/149)	468.17 (137.15 to 2941.20)	< 0.001
Management				
Emergency abdominal angiography				
(+)	2.9	(34/1189)	0.28 (0.18 to 0.44)	< 0.001
(−)	4.3	(102/2361)	Reference	–
3-year increase in time period	–	–	0.77 (0.65 to 0.91)	0.002

*OR* odds ratio, *CI* confidence interval, *ISS* Injury Severity Score, *AAST* American Association for the Surgery of Trauma

Table 4 summarizes the analysis of cases of isolated renal trauma. Patients with isolated renal trauma were significantly younger and showed a difference in the distribution of mechanisms of injury from those with multiple trauma. Falls down stairs, falls on the ground, and sports-related injuries were more frequent in the isolated renal trauma group. The proportion of nephrectomy did not differ between the groups. The median ISS of the isolated renal trauma group was significantly lower than that of the multiple trauma group (9 vs. 25, *P* < 0.001). The proportion of patients in shock on hospital arrival in the isolated renal trauma group was significantly lower than that of the multiple trauma group (3.7% vs. 12.7%, *P* < 0.001). The mortality rate in the isolated renal trauma group was significantly lower than that in the multiple trauma group (1.4% vs. 10.9%, *P* < 0.001).

**Table 4:** Patient characteristics of patients with isolated renal trauma and multiple trauma

	Isolated renal trauma		Multiple trauma		
Characteristics	*n* = 509		*n* = 3041		*P* value
Age, median, Q1–Q3	36	17–62	43	24–66	< 0.001
Age group, *n* (%)					< 0.001
< 20 years	155	(30.5)	452	(14.9)	
20–64 years	240	(47.2)	1777	(58.4)	
≥ 65 years	114	(22.4)	812	(26.7)	
Male sex, *n* (%)	368	(72.2)	2267	(74.5)	0.308
Mechanism, *n* (%)					< 0.001
Car accident	30	(5.9)	526	(17.3)	
Motorcycle accident	52	(10.2)	745	(24.5)	
Bicycle accident	37	(7.3)	234	(7.7)	
Pedestrian accident	6	(1.2)	347	(11.4)	
Fall from height	32	(6.3)	584	(19.2)	
Fall down stairs	79	(15.5)	244	(8.0)	
Fall on the ground	137	(26.9)	128	(4.2)	
Sports-related injury	93	(18.3)	52	(1.7)	
Others	43	(8.4)	181	(6.0)	
ISS, median, Q1–Q3	9	9–16	25	17–36	< 0.001
Shock on arrival, *n* (%)	18	(3.7)	387	(12.7)	< 0.001
AAST grade					< 0.001
I	81	(15.9)	1,327	(43.6)	
II	25	(4.9)	333	(11.0)	
III	238	(46.8)	801	(26.3)	
IV	147	(28.9)	449	(14.8)	
V	18	(3.5)	131	(4.3)	
Management, *n* (%)					
Emergency abdominal angiography	179	(35.2)	1,010	(33.2)	0.416
Nephrectomy	20	(3.9)	116	(3.8)	0.999
Mortality, *n* (%)	7	(1.4)	330	(10.9)	< 0.001

*ISS* Injury Severity Score, *AAST* American Association for the Surgery of Trauma

## Discussion

We reported the comprehensive analysis of the characteristics and management of cases with patients with blunt renal trauma, their temporal trends, and factors associated with patient outcomes using a nationwide database in Japan. Blunt renal trauma accounted for 1.0% of all blunt injuries registered in the JTDB, which was a similar rate to that reported in previous studies [17, 19]. As the number of institutions participating in the JTDB increased, the number of patients with blunt renal trauma increased. Patients of ≥ 65 years of age accounted for 26.1% of our total cohort, and this proportion increased significantly in each period. It may reflect the aging of the population in Japan, the change in the distribution of mechanisms of injury, and an increase in the number of institutions participating in the JTDB [20]. Although there is no apparent change in the median ISS, there was a decreasing trend in the percentage of patients in shock on hospital arrival. This may be partially due to changes in road safety regulations and behavioral patterns [21, 22]. Regarding the management of blunt renal trauma, we observed an increase in the use of emergency abdominal angiography and a decline in nephrectomy, which is a similar trend to Western countries [6, 7, 23]. The mortality rate decreased from 15.3 to 7.3% over 15 years, which is a similar trend to previous studies [24].

### Analysis of mortality

We report that factors such as age ≥ 65 years, shock on hospital arrival, concomitant injuries to the head/neck, pelvis/lower-extremity, liver, spleen, gastrointestinal tract, and AAST grades III, IV, and V were significantly associated with mortality. The gradient of mortality in each AAST grade was consistent with that in the National Trauma Data Bank in the USA [25]. We validated the association between the AAST grade and mortality in the Japanese population. Emergency abdominal angiography was associated with a lower mortality rate, which may reflect a benefit of non-operative management after angiography.

### Analysis of nephrectomy

Nephrectomy was more likely performed in those with shock on hospital arrival, concomitant splenic injury, and AAST grade ≥ III. Previous studies demonstrated the AAST grade, and other indications for laparotomy were associated with nephrectomy, which is consistent with our results [26, 27]. As high-grade renal trauma is associated with a higher risk of treatment failure in patients undergoing non-operative management in comparison to cases of lower-grade renal trauma, an appropriate assessment of the renal injury is important for selecting the appropriate management [16]. Patients with concomitant thoracic injury were less likely to receive nephrectomy, possibly because angiography and transcatheter arterial embolization (TAE) were less invasive and because it is relatively easy to control bleeding in cases involving trauma-induced coagulopathy and respiratory distress due to chest trauma [28–30].

### Isolated renal trauma

Isolated renal trauma happened in young people and was more frequent in patients who had experienced falls down stairs, falls on the ground, and sports-related injury, which may be damaged by a relatively small but localized force. A previous study showed that sports-related blunt renal trauma is more likely to occur in isolation without other abdominal or thoracic injury [31]. The mortality rate in patients with isolated renal trauma was 1.4%; however, we could not examine the cause of death.

### Limitations

The present study was associated with some limitations. First, although we analyzed a nationwide trauma database in which major critical care centers in Japan participated, the JTDB is not a population-based sample of trauma patients and the data are registered voluntarily. Therefore, selection and information biases both exist. Second, not all AIS codes correspond to AAST grades. However, the method to identify renal trauma has been successfully applied in multiple studies [15–17]. Third, because JTDB does not include data on TAE for renal injury and the failure of non-operative management, we could not assess these factors. Lastly, our results may not be fully applicable to other areas that have different healthcare systems, legislation, and age distribution of the population [21, 32, 33]. However, our results may be useful for improving trauma care in developed countries with an aging population.

## Conclusions

This study provides a comprehensive analysis of blunt renal trauma cases registered in a nationwide trauma database over a 15-year period. We demonstrated that the AAST grade and emergency angiography were associated with mortality and the need for nephrectomy in patients with blunt renal trauma in the Japanese population. By understanding the current trends in patient characteristics and management, as well as the factors associated with important clinical outcomes, our findings can help to improve the quality of care for patients with blunt renal trauma.

## Data Availability

The data that support the findings of this study are available from the JTDB, but the availability of these data is restricted.

## Abbreviations

AAST: American Association for the Surgery of Trauma
JTDB: Japan Trauma Data Bank
AIS: Abbreviated Injury Scale
ISS: Injury Severity Score
OR: Odds ratio
CI: Confidence interval
TAE: Transcatheter arterial embolization
